# Analysis of a multiple-quantum-dots embedded ring structure for potential optically-controlled quantum switch or spin filter

**DOI:** 10.1038/s41598-020-73275-x

**Published:** 2020-10-01

**Authors:** Zelong He, Xinwei Zhao, Kongfa Chen, Jiyuan Bai, Yong Guo

**Affiliations:** 1 School of Electronic and Information Engineering, Yangtze Normal University, Chongqing, 408003 People’s Republic of China; 2 Department of Physics, Tokyo University of Science, Tokyo, 162-8601 Japan; 3 College of Materials Science and Engineering, Fuzhou University, Fuzhou, Fujian 350108 People’s Republic of China; 4 Department of Physics and State Key Laboratory of Low-Dimensional Quantum Physics, Tsinghua University, Beijing, 100084 People’s Republic of China; 5 Collaborative Innovation Center of Quantum Matter, Beijing, People’s Republic of China

**Keywords:** Electronic properties and materials, Electronic devices

## Abstract

We theoretically study the average current through a ring embedded with multiple quantum dots in each arm subjected to a time-dependent external field. A current resonance band can be observed in a six-quantum-dot system. In the presence of a time-dependent external field, mutual transformation occurs between the resonance band and antiresonance band, indicating an effective optically-controlled quantum switch can be realized in a wider quantum dot’s energy regime. As the Zeeman effect is introduced, the conversion between 100 and − 100% for spin polarization $p$ can be realized by adjusting the frequency of time-dependent external field, suggesting a physical scheme of an optically-controlled spin filter. The present work sheds lights onto the design and quantum computation of future nano-devices.

## Introduction

Many different types of hardware for embodying qubits have been implemented for performing elementary quantum gate operations. These include dopants in semiconductors^1^, Josephson junctions^2^, and quantum dots^3^. In the past 20 years, qubits based on different degrees of electrons in quantum dots have been developed, including charge states of electrons^4^, spin states of an electron^5^, and other hybrid states^6^. Spintronics based on quantum dots and its applications on spin-functional devices have attracted broad scientific interests^7–10^. Quantum computing schemes based on the spin of quantum dots have been proposed^11^. It is expected that quantum dot devices will be realized as integrated quantum chips in the future.

Applying a time-dependent external field, such as a microwave or terahertz (THz) field, to quantum dot system can induce photon-assisted tunneling^12, 13^, which enables electrons to reach previously inaccessible energy states by absorbing or emitting photons. Research on photon-assisted tunneling is vital in basic industries and has attracted extensive interests^14, 15^. A THz detector via the THz photon-assisted tunneling through an InAs quantum dot has been proposed^16, 17^ and experimentally verified^18, 19^. The photon-assisted sideband derived from the transition between the ground state and the excited state can be well explained using the non-equilibrium Green's function method^20^, in good agreement with the experiment^21^. Photon-assisted tunneling provides as a useful approach for studying the coherent properties of charge qubits^22^.

In recent years, more attention has been paid to multiple quantum dots system, which provides the possibility of manipulating each quantum dot individually and increases the dimension of the parameters pace for the transmission characteristics. Therefore, in multiple quantum dots system there are some new transmission characteristics that do not exist in single- or double-quantum dot. Multiple quantum dots systems are often designed to obtain efficient spin filters^23–26^. Spin-polarized windows can be observed in a laterally coupled double-quantum-dot array^23^ and a side-coupled triple-quantum-dot array^24^. An efficient spin filter is achieved in a diamond-like multiple quantum dots device^25, 26^. Novel and interesting physical phenomena are expected when the time-dependent external field is applied to the multiple quantum dots system.

**Figure 1:**
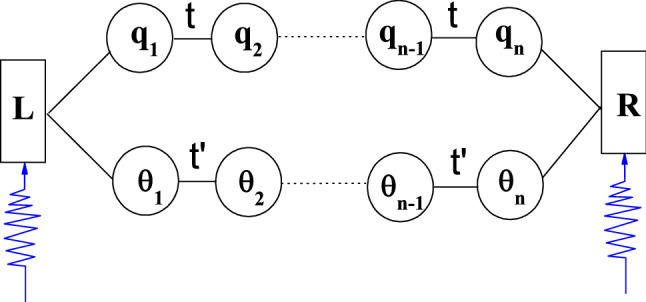
Schematic of a multiple-quantum-dots embedded ring structure.

Herein, we design a quantum ring, in which a pair of linearly connected multiple quantum dots arrays are embedded in the upper and lower arms (see Fig. 1). By using the Keldysh non-equilibrium Green's function technique^27^, we theoretically study the photon-assisted electron transport properties of the system. The average current of the system is effectively manipulated by adjusting the parameters such as the interdot coupling strengths, the amplitude and frequency of time-dependent AC external field.

## Model and theoretical methods

In order to understand the basic characteristics of time-dependent quantum transport, the inter-dot and intra-dot Coulomb interaction may be neglected. The DC bias voltage $V_{DC}$ and a time-dependent external field are applied to the two terminals of the ring. The time-dependent field $W_{L} (t) = W_{L} \cdot \cos (\omega t)$ and $W_{R} (t) = W_{R} \cdot \cos (\omega t)$. The lower corners marked with $L$ and $R$ represent the left and right leads, respectively; *W*_*L*(*R*)_ and $\omega$ are the amplitude and frequency of the time-dependent external field, respectively.

The Hamiltonian of the system can be written as.

1 $$\begin{gathered} H = \sum\limits_{\beta = L,\;R} {\sum\limits_{{\overset{\lower0.5em\hbox{$\smash{\scriptscriptstyle\rightharpoonup}$}} {k} ,\sigma }} {\varepsilon_{{\overset{\lower0.5em\hbox{$\smash{\scriptscriptstyle\rightharpoonup}$}} {k}_{\beta } }} } \left( t \right)c_{{\overset{\lower0.5em\hbox{$\smash{\scriptscriptstyle\rightharpoonup}$}} {k}_{\beta } \sigma }}^{ + } } c_{{\overset{\lower0.5em\hbox{$\smash{\scriptscriptstyle\rightharpoonup}$}} {k}_{\beta } \sigma }}^{{}} \hfill \\ + \sum\limits_{\sigma ,j = 1}^{n} {\left( {\varepsilon_{{q_{j} \sigma }} d_{{q_{j} \sigma }}^{ + } d_{{q_{j} \sigma }} + \varepsilon_{{\theta_{j} \sigma }} d_{{\theta_{j} \sigma }}^{ + } d_{{\theta_{j} \sigma }} } \right) + \sum\limits_{\sigma ,j = 1}^{n - 1} {\left( {td_{{q_{j} \sigma }}^{ + } d_{{q_{j + 1}\sigma }} + t_{{}}^{^{\prime}} d_{{\theta_{j} \sigma }}^{ + } d_{{\theta_{j + 1}\sigma }} + H.C.} \right)} } \hfill \\ + \sum\limits_{{\overset{\lower0.5em\hbox{$\smash{\scriptscriptstyle\rightharpoonup}$}} {k} \sigma }} {(t_{{q_{1} \sigma L}} c_{{\overset{\lower0.5em\hbox{$\smash{\scriptscriptstyle\rightharpoonup}$}} {k} L\sigma }}^{ + } d_{{q_{1} \sigma }} + t_{{\theta_{1} \sigma L}} c_{{\overset{\lower0.5em\hbox{$\smash{\scriptscriptstyle\rightharpoonup}$}} {k} L\sigma }}^{ + } d_{{\theta_{1} \sigma }} + t_{{q_{n} \sigma_R}} c_{{\overset{\lower0.5em\hbox{$\smash{\scriptscriptstyle\rightharpoonup}$}} {k} R\sigma }}^{ + } d_{{q_{n} \sigma }} + t_{{\theta_{n} \sigma_R}} c_{{\overset{\lower0.5em\hbox{$\smash{\scriptscriptstyle\rightharpoonup}$}} {k} R\sigma }}^{ + } d_{{\theta_{n} \sigma }} + H.c.)} \hfill \\ \end{gathered}$$

where the electron energy level $$\varepsilon_{{\overset{\lower0.5em\hbox{$\smash{\scriptscriptstyle\rightharpoonup}$}} {k}_{\beta } }} (t) = \varepsilon_{{\overset{\lower0.5em\hbox{$\smash{\scriptscriptstyle\rightharpoonup}$}} {k}_{\beta } }}^{0} + eV + eW_{\beta } (t) = \varepsilon_{{\overset{\lower0.5em\hbox{$\smash{\scriptscriptstyle\rightharpoonup}$}} {k}_{\beta } }}^{0} + eV + eW_{\beta } \cos (\omega t). c_{{\overset{\lower0.5em\hbox{$\smash{\scriptscriptstyle\rightharpoonup}$}} {k}_{\beta } \sigma }}^{ + } (c_{{\overset{\lower0.5em\hbox{$\smash{\scriptscriptstyle\rightharpoonup}$}} {k}_{\beta } \sigma }}^{{}} )$$ represents the creation (annihilation) operator for electrons in lead $$\beta$$ with spin index $$\sigma$$, and $$\overset{\lower0.5em\hbox{$\smash{\scriptscriptstyle\rightharpoonup}$}} {k}$$ is the wave vector. $$d_{{q_{j} \left( {\theta_{j} } \right)\sigma }}^{ + } (d_{{q_{j} \left( {\theta_{j} } \right)\sigma }} )$$ denotes the electron creation (annihilation) operator of the quantum dot with the energy level $$\varepsilon_{{q_{j} \left( {\theta_{j} } \right)\sigma }} = \varepsilon_{{q_{j} \left( {\theta_{j} } \right)}}^{0} + \sigma B$$, $$\varepsilon_{{q_{j} \left( {\theta_{j} } \right)}}^{0}$$ is the single particle energy level of the quantum dot $$q_{j} \left( {\theta_{j} } \right)$$, and $$B = g\mu_{B} H$$, in which $$g$$ and $$\mu_{B}$$ represent the Lander g-factor and Bohr magneton, respectively. $$t(t^{\prime } )$$ is the coupling strength between two adjacent quantum dots in each arm. $$t_{i\sigma L\left( R \right)}$$ describes the $$\overset{\lower0.5em\hbox{$\smash{\scriptscriptstyle\rightharpoonup}$}} {k}$$-independent coupling strength between the lead $$L\left( R \right)$$ and quantum dot $$i$$.

The time-dependent transient current $I\left( t \right)$ is determined by^19, 20^

2 $$I_{\beta \sigma } (t) = - \frac{2e}{\hbar }{\text{Im}} \int_{ - \infty }^{t} {dt^{\prime}} \int {\frac{d\varepsilon }{{2\pi }}Tr\left\{ {e^{{ - i\varepsilon (t^{\prime} - t)}} \Gamma_{\sigma }^{\beta } (\varepsilon ,t,t^{\prime})\left[ {G_{\sigma }^{ < } (t,t^{\prime}) + f_{\beta } (\varepsilon )G_{\sigma }^{r} (t,t^{\prime})} \right]} \right\}} ,$$

in which $f_{\beta } (\varepsilon ) = \left\{ {1 + \exp \left[ {{{\left( {\varepsilon - \mu_{\beta } } \right)} \mathord{\left/ {\vphantom {{\left( {\varepsilon - \mu_{\beta } } \right)} {k_{B} T}}} \right. \kern-\nulldelimiterspace} {k_{B} T}}} \right]} \right\}^{ - 1}$ reflects the Fermi distribution function, and the chemical potential can be expressed as *μ*_*L*_=−*μ*_*R*_ = *V*_DC_/2. The Green’s function $G^{ < } = G^{ r }\Sigma^{<} G^{ a }$, in which $\sum_{\beta \sigma }^{ < } = \sum\limits_{k} {t_{\beta \sigma }^{*} } g_{\beta \sigma k}^{ < } t_{\beta \sigma }^{{}}$ and $G^{ a }=(G^{ r })^{+}$.

In the wide-band limit, the relation between the retarded self-energy and the linewidth function is given by

3 $$\Sigma_{\beta \sigma }^{r} (t,t^{\prime}) = - \frac{i}{2}\delta (t - t^{\prime})\Gamma_{\sigma }^{\beta } ,$$

where $\Gamma_{{ll^{\prime}\sigma }}^{\beta } (\varepsilon ,t,t^{\prime}) = 2\pi \rho_{\beta \sigma } t_{l\sigma \beta } t_{{l^{\prime}\sigma \beta }}^{*} e^{{i\int_{{t^{\prime}}}^{t} {W_{\beta } (\tau )} d\tau }}$, and $\rho_{\beta \sigma }$ represents the spin density of state in the leads $\beta$.

Utilizing the Dyson equation, the retarded Green's function $G^{r}$ is achieved from the free Green’s function $g_{\sigma }^{r} \left( \varepsilon \right)$ of the quantum dots without couplings to the leads

4 $$G_{\sigma }^{r} (t,t^{\prime}) = \int {\frac{d\varepsilon }{{2\pi }}\exp \left[ { - i\varepsilon (t - t^{\prime})} \right]G_{\sigma }^{r} (\varepsilon )} ,$$

5 $$G_{\sigma }^{r} (\varepsilon ) = \left\{ {\left[ {g_{\sigma }^{r} (\varepsilon )} \right]^{{ - 1}} - \Sigma _{\sigma }^{r} (\varepsilon )} \right\}^{{ - 1}} .$$

Using the equation of motion method and the Dyson equation, we can calculate the retarded Green’s function

6 $$\left[ {G_{\sigma }^{r} \left( \varepsilon \right)} \right]^{ - 1} = \left( {\begin{array}{*{20}c} {G_{{q_{1} }} } & t & 0 & 0 & 0 & {\tau_{11} } & 0 & 0 & 0 & 0 \\ t & {\varepsilon - \varepsilon_{{q_{2} }} } & t & 0 & 0 & 0 & 0 & 0 & 0 & 0 \\ 0 & t & {\varepsilon - \varepsilon_{{q_{3} }} } & t & 0 & 0 & 0 & 0 & 0 & 0 \\ 0 & 0 & t & O & t & 0 & 0 & 0 & O & 0 \\ 0 & 0 & 0 & t & {G_{{q_{n} }} } & 0 & 0 & 0 & 0 & {\tau_{nn} } \\ {\tau_{11} } & 0 & 0 & 0 & 0 & {G_{{\theta_{1} }} } & {t^{\prime}} & 0 & 0 & 0 \\ 0 & 0 & 0 & 0 & 0 & {t^{\prime}} & {\varepsilon - \varepsilon_{{\theta_{2} }} } & {t^{\prime}} & 0 & 0 \\ 0 & 0 & 0 & 0 & 0 & 0 & {t^{\prime}} & {\varepsilon - \varepsilon_{{\theta_{3} }} } & {t^{\prime}} & 0 \\ 0 & 0 & 0 & O & 0 & 0 & 0 & {t^{\prime}} & O & {t^{\prime}} \\ 0 & 0 & 0 & 0 & {\tau_{nn} } & 0 & 0 & 0 & {t^{\prime}} & {G_{{\theta_{n} }} } \\ \end{array} } \right).$$

In Eq. (6), $\tau_{11}^{{}} = \frac{i}{2}\sqrt {\Gamma_{{q_{1} }}^{L} \Gamma_{{\theta_{1} }}^{L} }$, $\tau_{nn}^{{}} = \frac{i}{2}\sqrt {\Gamma_{{q_{n} }}^{R} \Gamma_{{\theta_{n} }}^{R} }$, $G_{{q_{1} }} = \varepsilon - \varepsilon_{{q_{1} }} + \frac{i}{2}\Gamma_{{q_{1} }}^{L}$, $G_{{q_{n} }} = \varepsilon - \varepsilon_{{q_{n} }} + \frac{i}{2}\Gamma_{{q_{n} }}^{R}$, $G_{{\theta_{1} }} = \varepsilon - \varepsilon_{{\theta_{1} }} + \frac{i}{2}\Gamma_{{\theta_{1} }}^{L}$, and $G_{{\theta_{n} }} = \varepsilon - \varepsilon_{{\theta_{n} }} + \frac{i}{2}\Gamma_{{\theta_{n} }}^{R}$, where $\Gamma_{l}^{\beta } \left( {l = q_{1} ,q_{n} ,\theta_{1} ,\theta_{n} } \right)$ is short for $\Gamma_{ll}^{\beta }$.

The time-dependent transient current can be achieved by substituting Eq. (4) into Eq. (2)

7 $$I_{{\beta \sigma }} (t) = - \frac{e}{\hbar }\int {\frac{{d\varepsilon }}{{2\pi }}} Tr\text{Im} \left\{ {2f_{\beta } (\varepsilon )\Gamma _{\sigma }^{\beta } A_{{\beta \sigma }} (\varepsilon ,t) + i\Gamma _{\sigma }^{\beta } \sum\nolimits_{{\sigma = L,R}} {f_{\alpha } (\varepsilon )} A_{{\alpha \sigma }} (\varepsilon ,t)\Gamma _{\sigma }^{\alpha } A_{{\alpha \sigma }}^{ + } (\varepsilon ,t)} \right\}$$

in which

8 $$A_{\beta \sigma } \left( {\varepsilon ,t} \right) = \exp \left[ {\frac{{ie\left( {W_{\beta } } \right)\sin \left( {\omega t} \right)}}{\omega }} \right]\sum\limits_{\chi } {J_{\chi } \left( {\frac{{W_{\beta } }}{\omega }} \right)e^{in\omega t} G_{\sigma }^{r} \left( {\varepsilon_{\chi } } \right)}$$

In Eq. (8), $J_{\chi }$ is the first kind of Bessel function, and $\varepsilon_{\chi } = \varepsilon { - }\chi \omega$. The instantaneous current $I_{\beta\sigma}(t)$ can be obtained by numerical resolving Eq. (7). Therefore, the time-average current $<I>$ can be achieved by the following equation

9 $$\left\langle I \right\rangle = \frac{2e}{\hbar }\int {\frac{d\varepsilon }{{2\pi }}\sum\limits_{\chi } {Tr} } \left\{ {\left[ {J_{\chi }^{2} \left( {\frac{{W_{L} }}{\omega }} \right)f_{L} \left( \varepsilon \right) - J_{\chi }^{2} \left( {\frac{{W_{R} }}{\omega }} \right)f_{R} \left( \varepsilon \right)} \right]\Gamma_{\sigma }^{L} G_{\sigma }^{r} \left( {\varepsilon_{\chi } } \right)\Gamma_{\sigma }^{R} G_{\sigma }^{a} \left( {\varepsilon_{\chi } } \right)} \right\}$$

## Numerical results

Now we study the time-dependent quantum transmission characteristics of a multiple-quantum-dots embedded ring structure. Herein the DC bias is assumed as $V_{DC} = 0.05$, the temperature is taken as $k_{B} T = 0.001$, and the dot-lead coupling strength is set to $\Gamma^{\beta } = 0.25\Gamma_{0}$, with $\Gamma_{0}$ as the energy unit. The interdot coupling strengths $t = t_{{}}^{^{\prime}} = {{\sqrt 2 S} \mathord{\left/ {\vphantom {{\sqrt 2 S} 2}} \right. \kern-\nulldelimiterspace} 2}$, the energy levels of quantum dot $\varepsilon_{{q_{j} }}^{0} = \varepsilon_{{\theta_{j} }}^{0} = \varepsilon_{d}$, $\hbar = 1.0$ and $e = 1.0$. The energy levels of quantum dots can be more easily adjusted manually though the gate voltage. Therefore, we study the transmission coefficient and the spin polarization with respect to the energy levels of quantum dots.

**Figure 2:**
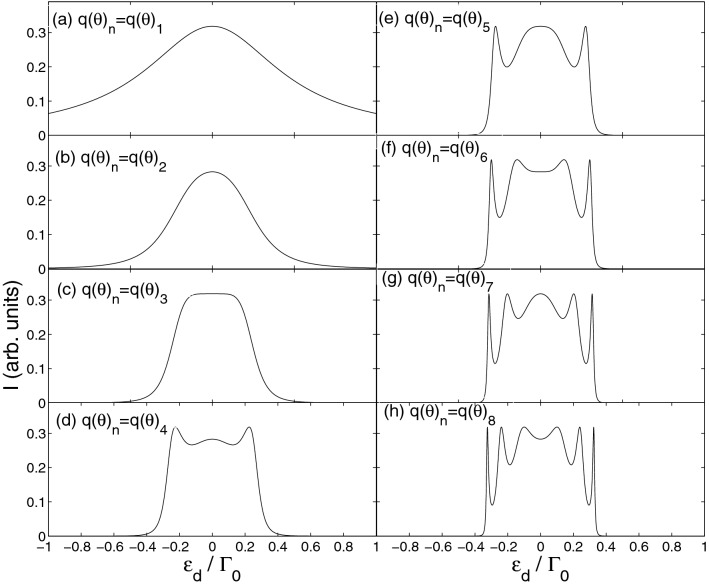
Current spectra versus quantum dot energy for several different structures. The relative parameters $\Gamma^{\beta } = 0.25\Gamma_{0}$, $W_{L\left( R \right)} = 0$, $B = 0$ and $S = 0.25$.

First we examine the current through several different structures $n\left( {1 - 8} \right)$ in the absence of time-dependent external field, as illustrated in Fig. 2. As $n = 1,2$, only one current peak emerges in the spectra. This indicates the energy levels are degenerated, due to the spatial symmetry of quantum dot structures. The current through the system with $n = 2$ is smaller than that through the system with $n = 1$ in the whole energy regime. This implies that it is more difficult for electrons to pass through the system with $n = 2$. An interesting phenomenon is that a current resonance band appears near the energy level $\varepsilon_{d} = 0$ as $n = 3$. When $n \ge 4$, the number of quantum dots increases, making the tunneling path of electrons complex, and thus leads to the appearance of multi-current peaks structure in the energy spectra. One can find a rule that when $n$ ($n \ge 5$) is odd, a current resonance peak emerges at the location of $\varepsilon_{d} = 0$, and in the case of $n + 1$, the current resonance peak splits to form a valley. Based on the above discussion and analysis, we focus on a systematic analysis of the system with $n = 3$, namely a six-quantum-dot system. This is because the current resonance band means a steady current, which makes it easier to control the electronic transport properties through the system.

**Figure 3:**
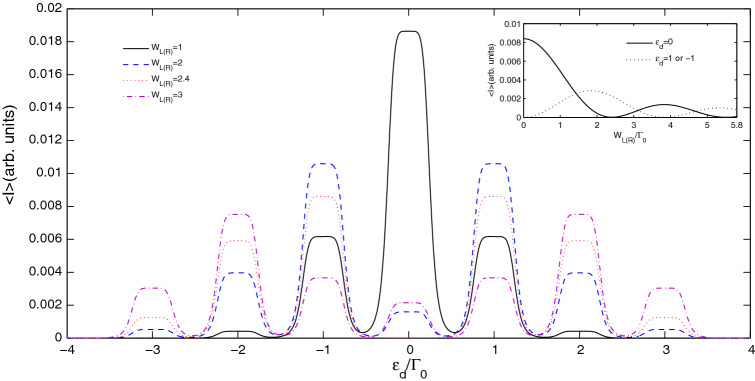
Average current spectra versus quantum dot energy at various amplitudes of symmetrical time-dependent field. The typical parameters are $S = 0.25$, $\omega = 1.0$, $n = 3$ and $B = 0$.

Figure 3 illustrates the average current spectra of the six-quantum-dot ring system versus the energy level of quantum dots for various amplitudes of the time-dependent external field. First, we focus on the average current spectrum in the case of $W_{L\left( R \right)} = 1.0$. The center of the main resonance band locates at the energy level $\varepsilon_{d} = 0$. A series of accompanying side-resonance bands emerge on either side of the main resonance band. As the amplitude of the time-dependent external field increases ($W_{L\left( R \right)} < 2.4$), the value of the main resonance band at the energy level $\varepsilon_{d} = 0$ in the current spectrum decreases substantially. However, the side-resonant bands in the average current spectrum oscillate out of phase with the main resonance band. For example, the value of the side-resonance band at the center of the energy level $\varepsilon_{d} = \pm 1,\; \pm 2$ grows larger. The reason is that the electron tunneling through the system exchanges the energy of $\chi \hbar \omega$ with the time-dependent external fields and results in new inelastic tunneling channels in the quantum dot, in which the transmission probabilities through the channels of $\varepsilon_{d} = 0$ are shared and suppressed. It is important to point out that when the amplitude of the time-dependent external field exceeds the threshold ($W_{L\left( R \right)} = 2.4$), the value of the main resonance band increases again with the increase of amplitude. This indicates that all the main resonant band and the associated side-resonant bands show oscillation effects, mainly due to the fact that the height of the $\chi$-order current resonant band is determined by the Bessel function, which is derived from the action of the time-dependent external field. A novel phenomenon is that as the amplitude $W_{L\left( R \right)} = 2.4$, the main resonance band is transformed into a wider antiresonance band. This implies that the mutual transformation of resonance band and antiresonance band can be realized by adjusting the amplitude of time-dependent external field. Based on this property, the system can be used as an effective optically-controlled quantum switch in a wider energy area. The inset in Fig. 3 shows the influence of the amplitude $W_{L\left( R \right)}$ from 0 to 5 on average current. Here the height of main resonance band changes like a cosine or sine relation with the increase of $W_{L\left( R \right)}$. We ascribe the phenomena to the counteracting effect between the external fields on the two leads. Moreover, one can find that a current zero point emerges at around $W_{L\left( R \right)} = 2.4$ as $\varepsilon_{d} = 0$. For $\varepsilon_{d} = \pm 1$, a current zero point can also be observed at around $W_{L\left( R \right)} = 3.9$. This indicates that the current zero point can be obtained for different values of $\varepsilon_{d}$. Therefore, an effective optically-controlled quantum switch can be easily realized in experiment by tuning the energy levels of quantum dots $\varepsilon_{d}$ or the amplitude of time-dependent external field $W_{L\left( R \right)}$.

**Figure 4:**
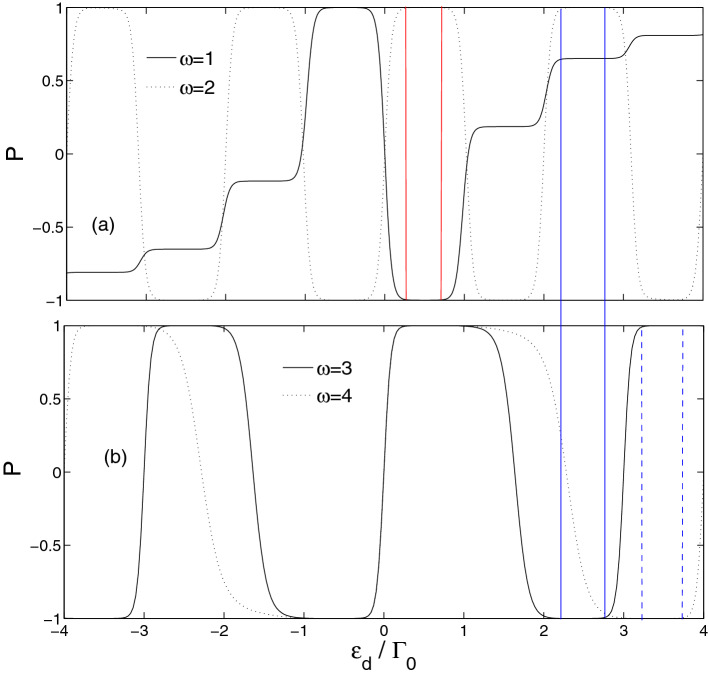
Spin polarization $p$ versus quantum dot energy at several different frequencies of time-dependent external field. The relative parameters are $S = 0.25$, $W_{L\left( R \right)} = 2.4$, $B = 0.2$, and $n = 3$.

Now we discuss the influence of time-dependent external field on the spin polarization. When the Zeeman effect is considered, all the energy levels of quantum dots grow into $\varepsilon_{j \uparrow } = \varepsilon_{d} + B$ and $\varepsilon_{j \downarrow } = \varepsilon_{d} - B$. The average currents corresponding to spin-up and -down will separate from each other, indicating the average current of the system is spin polarized. Figure 4 illustrates the dependence of spin polarization $p = {{\left( {\left\langle {I_{ \uparrow } } \right\rangle - \left\langle {I_{ \downarrow } } \right\rangle } \right)} \mathord{\left/ {\vphantom {{\left( {\left\langle {I_{ \uparrow } } \right\rangle - \left\langle {I_{ \downarrow } } \right\rangle } \right)} {\left( {\left\langle {I_{ \uparrow } } \right\rangle + \left\langle {I_{ \downarrow } } \right\rangle } \right)}}} \right. \kern-\nulldelimiterspace} {\left( {\left\langle {I_{ \uparrow } } \right\rangle + \left\langle {I_{ \downarrow } } \right\rangle } \right)}}$ on the energy levels of quantum dots for several different frequencies of the time-dependent external field. Herein, an external magnetic field on the quantum dots is taken as $B = 0.2$. The solid line in Fig. 4a represents the case of frequency $\omega = 1.0$. The spin polarization curve is antisymmetric with respect to the origin of coordinates, i.e., $\left( {0,0} \right)$. Therefore, we restrict our discussion in the positive energy level regime. The curve displays three steps, which originates from the appearance of a series of side-resonance bands corresponding to different photon-assisted tunneling processes. With increasing the energy levels of quantum dots, the spin polarization grows larger. In the case of $\omega = 2.0$(Fig. 4a), one can find many bands corresponding to the spin polarization $p = 100\%$ or $p = - 100\%$. Consequently, a completely spin-polarized average current can be implemented by tuning the energy levels of quantum dots through the gate voltages. This implies the spin up (down) electrons can tunnel through the system, while the tunneling of the spin down (up) electrons is prohibited. This strongly indicates that such a six-quantum-dot system can be applied as a spin filter. It is more intriguing and important that such a six-quantum-dot system can be realized as an optically-controlled spin filter by adjusting the frequency of the time-dependent external field. For the energy region surrounded by the two red vertical lines, the spin polarization $p = - 100\%$ and $p = 100\%$ as $\omega = 1.0$ and $\omega = 2.0$, respectively. This indicates the direction of the spin-polarized current can be changed by tuning the frequency of the time-dependent external field. The results demonstrate that the system can be applied as an optically-controlled spin filter. If the frequency $\omega$ further increases to 3 and 4, as shown in Fig. 4b, the range of energy levels corresponding to the spin polarization $p = - 100\%$ or $p = 100\%$ is expanded. By comparing the dot line in Fig. 4a with the solid line in Fig. 4b, for the energy region surrounded by the two blue vertical solid lines, it can be found the spin polarization $p = 100\%$ and $p = - 100\%$ as $\omega = 2.0$ and $\omega = 3.0$, respectively. By adjusting $\omega = 3.0$ or $\omega = 4.0$, spin polarization $p = 100\%$ or $p = - 100\%$ in the energy region surrounded by the two blue vertical dashed lines. This means that the system can be used as an efficient optically-controlled spin filter in more energy regions as long as the appropriate frequencies are chosen.

## Conclusions

In conclusion, the numerical results show that a six-quantum-dot ring is the best quantum device to be easily regulated. By adjusting the amplitude of time-dependent external field, the six-quantum-dot ring device can be used as an effective optically-controlled quantum switch in a wider energy regime, which can be easily realized by experiment. If the Zeeman effect is considered, this six-quantum-dot ring device can be applied as an optically-controlled spin filter by adjusting the frequency of time-dependent external field. It is stressed that this six-quantum-dot-molecule ring is a practical design to realize a nano-device possessing multiple functions including the optically-controlled quantum switch and spin filter.
